# Erratum to: Evaluation of the Safety, Tolerability, and Pharmacokinetics of Gammaplex® 10% Versus Gammaplex® 5% in Subjects with Primary Immunodeficiency

**DOI:** 10.1007/s10875-017-0418-2

**Published:** 2017-07-17

**Authors:** Richard L. Wasserman, Isaac R. Melamed, Mark R. Stein, Stephen Jolles, Miranda Norton, James N. Moy, for the GMX07 Study Group

**Affiliations:** 1 Allergy Partners of North Texas Research, Dallas, TX USA; 2 IMMUNOe International Research Centers, Centennial, CO USA; 3 Allergy Associates of the Palm Beaches, North Palm Beach, FL USA; 4 Immunodeficiency Centre for Wales, University Hospital of Wales, Cardiff, UK; 5 Bio Products Laboratory Ltd., Dagger Lane, Elstree, Hertfordshire, UK; 6 Section of Allergy and Immunology, Department of Immunology and Microbiology, Rush University Medical Center, Chicago, IL USA

**Erratum to: J Clin Immunol** (**2017) 37:301–310.**

**DOI 10.1007/s10875-017-0383-9**

The article Evaluation of the Safety, Tolerability, and Pharmacokinetics of Gammaplex® 10% Versus Gammaplex® 5% in Subjects with Primary Immunodeficiency, written by Richard L. Wasserman, Isaac R. Melamed, Mark R. Stein, Stephen Jolles, Miranda Norton, James N. Moy and for the GMX07 Study Group, was originally published Online First without open access. After publication in volume 37, issue 3, page 301–310 the author decided to opt for Open Choice and to make the article an open access publication. Therefore, the copyright of the article has been changed to © The Author(s) 2017 and the article is forthwith distributed under the terms of the Creative Commons Attribution 4.0 International License (http://creativecommons.org/licenses/by/4.0/), which permits use, duplication, adaptation, distribution and reproduction in any medium or format, as long as you give appropriate credit to the original author(s) and the source, provide a link to the Creative Commons license, and indicate if changes were made.

